# Withdrawal of Colistin Reduces Incidence of *mcr-1*-Harboring IncX4-Type Plasmids but Has Limited Effects on Unrelated Antibiotic Resistance

**DOI:** 10.3390/pathogens10081019

**Published:** 2021-08-12

**Authors:** Zunfang Tu, Ju Gu, Haoyu Zhang, Jinxin Liu, Junrui Shui, Anyun Zhang

**Affiliations:** 1 Animal Disease Prevention and Food Safety Key Laboratory of Sichuan Province, Key Laboratory of Bio-Resource and Eco-Environment of Ministry of Education, College of Life Sciences, Sichuan University, Chengdu 610065, China; 2019222040119@stu.scu.edu.cn (Z.T.); guju2018@gmail.com (J.G.); hoyeezhang@hotmail.com (H.Z.); 2019222040090@stu.scu.edu.cn (J.S.); 2 Laboratory of Gastrointestinal Microbiology, College of Animal Science and Technology, Nanjing Agricultural University, Nanjing 210095, China; jxnliu@njau.edu.cn

**Keywords:** ban of colistin, *mcr-1*, IncX4-type plasmids, *Escherichia coli*, *Klebsiella pneumoniae*

## Abstract

The global dissemination of plasmid-mediated colistin resistance gene *mcr* and its variants have posed a great threat to public health. Therefore, the Chinese government banned the use of colistin as a feed additive in livestock in April 2017. To explore the dynamic changes of overall antibiotic resistance genes (ARGs) and phylogenetic relationship of bacteria from a single pig farm before and after the withdrawal of colistin, fecal swab samples were collected from a large-scale pig farm before (*n* = 32; 2 months pre-withdrawal of colistin) and after withdrawal of colistin (*n* = 30; 13 months post-withdrawal of colistin). *Escherichia coli* and *Klebsiella pneumoniae* were isolated. Whole-genome sequencing (Illumina, MiSeq) was performed to examine ARGs, plasmids and the genetic relationship of the isolates. The overall SNP results indicated all isolates had high genetic diversity, and the evolutionary relationship across isolates was not influenced by the ban of colistin. However, the prevalence of *mcr-1.1* (5.6%, *p* < 0.01) was significantly lower than before the ban (86.4%). Plasmid profiling analysis showed that 17 of 20 (85.0%) observed *mcr-1.1* genes reside on IncX4-type plasmids, 16 of which (94.1%) were from isolates before the ban. On the contrary, the presence of *bla*_CTX-M_ gene was significantly increased (*p* = 0.0215) post-withdrawal of colistin. Our results showed that withdrawal of colistin reduced the incidence of *mcr-1*-harboring IncX4-type plasmids, but had limited influences on unrelated ARGs.

## 1. Introduction

Antimicrobial resistance has been a pressing concern to public health and the recent prevalence of carbapenem-resistant Enterobacteriaceae (CRE) certainly complicated this issue [1]. In human medicine, colistin has been considered as the last defense against highly resistant Gram-negative bacteria, especially carbapenem-resistant pathogens [2]. Colistin was widely used in food animal production as an animal growth promoter, exerting selective pressure on bacteria for gaining colistin resistance. As a result, *mcr-1*, the first plasmid-mediated colistin resistance gene, was initially reported in 2016 [3], and was found worldwide across multiple bacterial species residing in transferable plasmids including IncX4, IncI2, IncP, and IncHI2 [4,5]. To tackle this issue, the Chinese government has banned the use of colistin as a feed additive in livestock since April 2017 [6].

Yang Wang [7] and Cong Shen [8] recently reported the dramatic reduction of the prevalence of *mcr-1* in *Escherichia coli* and investigated the genomic epidemiology in *mcr-1*-positive *E. coli* from both animals and humans after the ban of colistin. While comprehensive, these studies did not analyze the prevalence of other transferable resistance genes in the same farm before and after the withdrawal of colistin, and such influences remain unclear. Therefore, the present study tracked the incidence of all measurable acquired resistance in both *E. coli* and *Klebsiella pneumoniae* from a pig farm during the policy change.

## 2. Materials and Methods

### 2.1. Sample Collection, Bacteria Isolates and Colistin Susceptibility Testing

Fecal swabs were collected from different pigs (*n* = 62) in a breeding pig farm (more than 3000 pigs) in February 2017 (*n* = 32; 2 months pre-withdrawal of colistin) and May 2018 (*n* = 30; 13 months post-withdrawal of colistin) in Sichuan, China. Compared with 2017, the general antibiotics usage (such as beta-lactams, aminoglycosides, sulfonamides, tetracyclines, macrolides, quinolones, and florfenicol) in 2018 was not changed except that colistin was banned for about 13 months. All samples were collected with sterile sampling tubes, placed on ice, and transported to the laboratory (Sichuan University, Chengdu, Sichuan, China) for immediate processing. Fecal swabs were cultured in brain–heart infusion (BHI) broth for 24 h at 37 °C, and then streaked on blood agar and MacConkey agar plates without adding any selective antibiotics. *E. coli* and *K. pneumoniae* strains were isolated on MacConkey agar and blood agar plates, respectively. To avoid duplicate strains, one strain was selected from each plate. No more than one *E. coli* or *K. pneumoniae* was isolated per fecal swab (Table S1). All the isolates were purified by subculturing, identified via an automated system (BD Diagnostic Systems, Sparks, MD, USA) and further confirmed by 16S rRNA sequencing [9]. According to the guidelines of the Clinical and Laboratory Standards Institute (CLSI) [10,11], the minimum inhibitory concentration (MIC) of coslistin for *E. coli* and *K. pneumoniae* was determined by broth micro-dilution method with *E. coli* ATCC25922 (purchased from the American Type Culture Collection (ATCC), Manassas, VA, USA) as the quality control bacteria. All isolates were tested in triplicate.

### 2.2. WGS and Sequence Analysis

To systematically characterize the ARGs profile changes after ban of colistin, the whole genome DNA of *E. coli* and *K. pneumoniae* isolates was extracted using a Tiangen genomic DNA kit (Tiangen, China). Then, whole-genome sequencing (WGS) was performed on the Illumina MiSeq platform (150 paired-end, 200 X coverage). FASTQ reads were quality trimmed using Trimomatic [12], with bases with PHRED scores of <30 removed from the trailing end. If the read length post trimming was less than 50 bp, the read and its pair were discarded. The sequencing reads were assembled via SPAdes (v3.13.1) [13]. Resistance and plasmid profiles in assembled genomes were identified through Resfinder (v.3.2) [14] with 90% identity and PlasmidFinder (v.2.0) [15]. The changes of ARGs number per bacterial genome before and after colistin withdrawal were analyzed using the Wilcoxon test and the P values were adjusted with Bonferroni correction. Multi-Locus Sequence Typing was performed using the MLST database (https://cge.cbs.dtu.dk/services/MLST/ (accessed on 16 March 2021)) according to genetic variations of seven housekeeping genes (*Klebsiella pneumoniae*: *gapA*, *infB*, *mdh*, *pgi*, *phoE*, *ropB*, and *tonB*; *Escherichia coli*: *adk*, *fumC*, *gyrB*, *icd*, *mdh*, *recA* and *purA*) [16,17]. Single nucleotide polymorphism (SNP) phylogenetic analysis was performed using CSI Phylogeny (v1.4) [18], *E. coli* strain UM146 N315 (NC_017632.1), and *K. pneumoniae* strain PMK1 (NZ_CP008929.1) were used as the reference and rooting genomes. Phylogenetic trees were visualized with MEGA7 and Evolview [19]. In addition, BRIG software was used to make a circular map of the plasmid genome.

### 2.3. Data Availability

The nucleotide sequences of bacterial genomes of *E. coli* (*n* = 23) and *K. pneumoniae* isolates (*n* = 17) were deposited in the NCBI database and are publicly available under accession number PRJNA752000. 

## 3. Results and Discussion

### 3.1. Bacterial Isolation, MLST and SNP Phylogenetic Analyses

**Figure 1:**
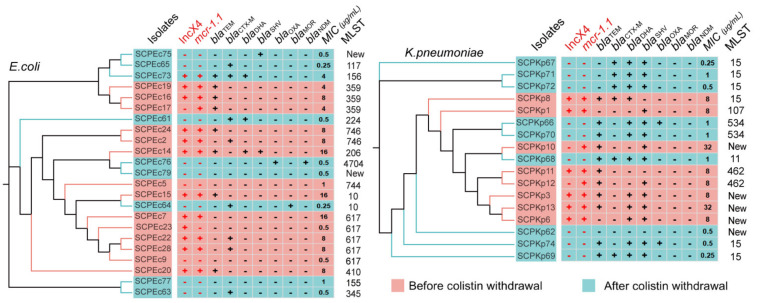
Single nucleotide polymorphism (SNP) phylogenetic analysis of E. coli (Ec) and *K. pneumoniae* (Kp) and the presence of *mcr-1.1*, IncX4-type plasmid, beta-lactams resistance genes, minimum inhibitory concentration (MIC) of colistin (μg/mL) and ST during policy change. New stands for new ST (Tables S2 and S3). The two columns of *mcr-1.1* and IncX4-type plasmid were highlighted in red, indicating that they were related. *bla*_TEM_ included variants encoding TEM-116,-181,-1A,-1B,-1C; *bla*_CTX-M_ included variants encoding CTX-M-3,-14,-55,-65; *bla*_DHA_ included variants encoding DHA-1,-4; *bla*_SHV_ variants were not differentiated; *bla*_OXA_ encodes OXA-1; *bla*_MOR_ encodes MOR-2; and *bla*_NDM_ encodes NDM-1 (Table S4).

A total of 22 (14 *E. coli* and 8 *K. pneumoniae*) and 18 isolates (9 *E. coli*, and 9 *K. pneumoniae*) were collected before and after the withdrawal of colistin, respectively. In addition, 5 *Acinetobacter baumannii*, 16 *Morganella morganii*, and 18 *Proteus mirabilis* were also isolated on MacConkey agar, and 6 *Enterococcus faecalis* were isolated on blood agar (Table S1). SNP phylogenetic analysis classified 23 *E. coli* and 17 *K. pneumoniae* isolates into 16 and 12 clusters, respectively, indicating that isolates had high genetic diversity, and evolutionary relationship across isolates was not influenced by the ban of colistin (Figure 1). MLST analysis indicated that the predominant STs for *E. coli* were ST617 and ST359 before withdrawal, which partly agreed with another survey of *mcr-1*-positive *E. coli* isolates from production animals in Poland [20]. Moreover, no predominant ST clades after withdrawal were observed (Figure 1 and Table S2), and ST10 was present before and after withdrawal of colistin. In addition to their presence in humans [7], ST206, ST10, ST410, and ST155 were observed in pigs, indicating potential transmission between livestock and humans. We observed no predominate ST clades for *K. pneumoniae* before withdraw of colistin, but ST15 was the predominant ST post-ban (Figure 1 and Table S3). ST15 is one of the emerging international high-risk clones causing nosocomial outbreaks worldwide [21,22], associated with a range of beta-lactamases, including OXA-48-like [23], NDM [24] and CTX-M [25]. This data indicated that the pig farm should strengthen surveillance and comprehensive infection control measures.

### 3.2. Overall ARG Profile Changing after Ban of Colistin

**Figure 2:**
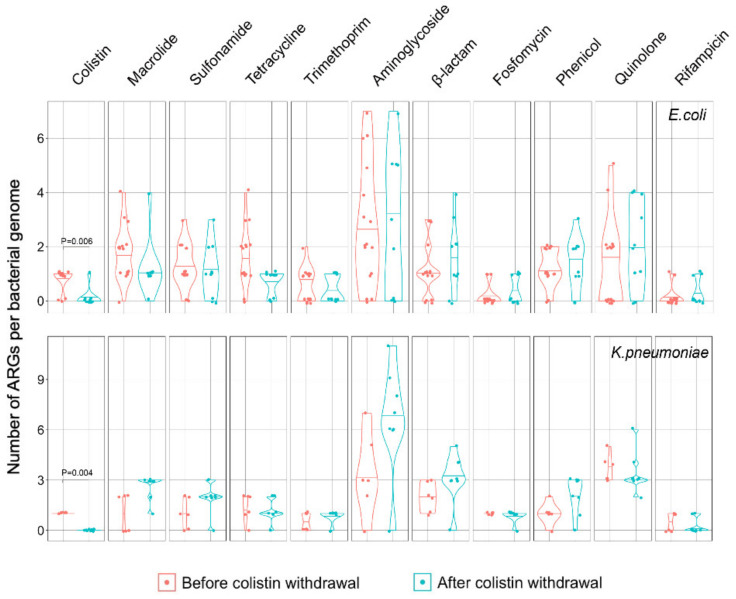
Violin plot depicting the prevalence of antibiotic resistance genes (ARGs) in *E. coli* and *K. pneumoniae* before (red color) and after (blue color) the withdrawal of colistin. Median number of ARGs was denoted as the horizontal line inside violin. Data were analyzed via a Wilcoxon test, and *p* values were adjusted by Bonferroni correction.

Resfinder-based [26] analysis indicated that the overall multidrug resistance profile remained relatively stable between sampling except for a dramatic reduction of the prevalence of *mcr-1.1* post-withdrawal of colistin (Figure 2 and Table S4). The prevalence of *mcr-1.1* after the withdrawal (one [5.6%] of 18) was significantly lower than that before the withdrawal (*E. coli*, *p* = 0.006, and *K. pneumoniae*, *p* = 0.004) (Figure 2). Similarly, Cong Shen et al. reported that after the ban, *mcr-1* prevalence decreased significantly in national pig farms, and a similar decrease occurred in samples from most sources in Guangzhou [8]. Plasmid profiling analysis [15] showed that 17 of 20 (85.0%) observed *mcr-1.1* genes reside on IncX4-type plasmids (Table S5 and Figure S1), 16 of which (94.1%) were from isolates before the ban (Figure 1), while only one *mcr-1.1*-harboring IncX4-type plasmid in China was found after the ban. Interestingly, the IncX4-type plasmids only carried the *mcr-1.1* gene and did not carry any other ARGs. These results indicated that the reduction of *mcr-1.1* was likely because of the loss of IncX4-type plasmids, the dominant *mcr-1.1* harboring plasmid in China [6]. This finding is consistent with the argument that mitigating the prevalence of *mcr-1* gene via cessation of colistin use is feasible [27].

Then, we further investigated whether and how the ban of colistin influenced the prevalence of other ARGs. No overall significant changes were observed in the prevalence of other ARGs classified into 10 classes of antibiotics between sampling (Figure 2). Conversely, the presence of *bla*_CTX-M_ gene was significantly increased (*p* = 0.0215) post-withdrawal of colistin. The probable reason may be the constant use of beta-lactam antibiotics on this farm. More worryingly, the *bla*_NDM-1_ gene carried by the IncR plasmid was detected in *E. coli* SCPEc76 after the ban (Figure 1), which was not observed before in the farm. The *bla*_NDM-1_ gene encodes the metallo-beta-lactamase NDM-1, which is a frequently occurring carbapenemase in *E. coli* and *K. pneumoniae* worldwide [28]. The emergence and spread of NDM-1-producing isolates in both humans and the environment have been reported in many countries, posing a serious threat to successful antibiotic therapy [29]. Moreover, IncR and IncHI1B plasmid carried many beta-lactam resistance genes, which were common plasmid types of *K. pneumoniae* [30,31]. After withdrawal of colistin, the prevalence of IncR and IncHI1B plasmid increased from 18.2% to 50.0% and from 1.1% to 44.4%, respectively, indicating that they may carry or even promote the transmission of beta-lactam resistance (Table S5). Our results also showed that the continuous use of beta-lactam antibiotics may provide constant selective pressure for beta-lactam resistance gene-related plasmids and increase the frequency of transmission of these plasmids. Importantly, we found that the ban of colistin has limited influence on resistance to antibiotics of other classes. Additional surveillance and policies against individual classes of antibiotics would be needed.

## 4. Conclusions

This study examined the dynamic changes of overall ARG profile and the phylogenetic relationship of bacteria from a single pig farm before and after the withdrawal of colistin. Our research demonstrated the overall ARG profile remained relatively stable between sampling except a dramatic reduction of the prevalence of *mcr-1.1* post-withdrawal of colistin, and such reduction was mainly linked to the loss of IncX4-type plasmids. However, we observed occurrences of beta-lactamase genes *bla*_CTX-M_ and *bla*_NDM__-1_ increased after the withdrawal of colistin, which are likely a result of the continuous use of beta-lactam antibiotics. The ban of colistin has limited influence on the prevalence of unrelated antibiotic resistance. Continued resistance surveillance and policies against other antibiotics on animal farms are needed.

## Data Availability

The nucleotide sequences of bacterial genomes of E. coli (n = 23) and K. pneumoniae isolates (n = 17) were deposited in the NCBI database and are publicly available under accession number PRJNA752000.

## Supplementary Materials

The following are available online at https://www.mdpi.com/article/10.3390/pathogens10081019/s1, Figure S1: Genome map of the plasmid pSCsl19-IncX4. Table S1: Bacterial isolates from 62 pig feces samples. Table S2: Detailed information on detected MLST alleles of 23 Escherichia coli isolates from pig feces. Table S3: Detailed information on detected MLST alleles of 17 Klebsiella pneumoniae isolates from pig feces. Table S4: Detected antibiotic resistance genes in genome sequences of 17 Klebsiella pneumoniae and 23 Escherichia coli isolates from pig feces. Table S5: Detected replicon types in genome sequences of 17 Klebsiella pneumoniae and 23 Escherichia coli isolates from pig feces.
