# Supplementary material for: Withdrawal of Colistin Reduces Incidence of mcr-1-Harboring IncX4-Type Plasmids but Has Limited Effects on Unrelated Antibiotic Resistance
Source: Pathogens. 2021 Aug 12;10(8):1019. doi: 10.3390/pathogens10081019 (PMC8398929; doi:10.3390/pathogens10081019)
Supplement: Supplementary file 1 [file pathogens-10-01019-s001.zip › pathogens-1273897 - Figure S1 (xml).docx]

Article

Withdrawal of Colistin Reduces Incidence of *mcr-1*-Harboring IncX4-Type Plasmids But Has Limited Effects on Unrelated Antibiotic Resistance

Zunfang Tu ^1†^, Ju Gu ^1†^, Haoyu Zhang ^1^, Jinxin Liu ^2^, Junrui Shui ^1^ and Anyun Zhang ^1*^

^1^ Animal Disease Prevention and Food Safety Key Laboratory of Sichuan Province, Key Laboratory of Bio-Resource and Eco-Environment of Ministry of Education, College of Life Sciences, Sichuan University, Chengdu 610065 , Sichuan, China; 2019222040119@stu.scu.edu.cn (Z.T.); guju2018@gmail.com (J.G.); hoyeezhang@hotmail.com (H.Z.); 2019222040090@stu.scu.edu.cn (J.S.)

^2^ Laboratory of Gastrointestinal Microbiology, College of Animal Science and Technology, Nanjing Agricultural University, Nanjing 210095, Jiangsu, China; [jxnliu@njau.edu.cn](mailto:jxnliu@njau.edu.cn)

† Zunfang Tu and Ju Gu contributed equally to this work.

***** Correspondence: [zhanganyun@scu.edu.cn](mailto:zhanganyun@scu.edu.cn); Tel: +86-28-8547-1599. Fax: +86-28-8547-1599.


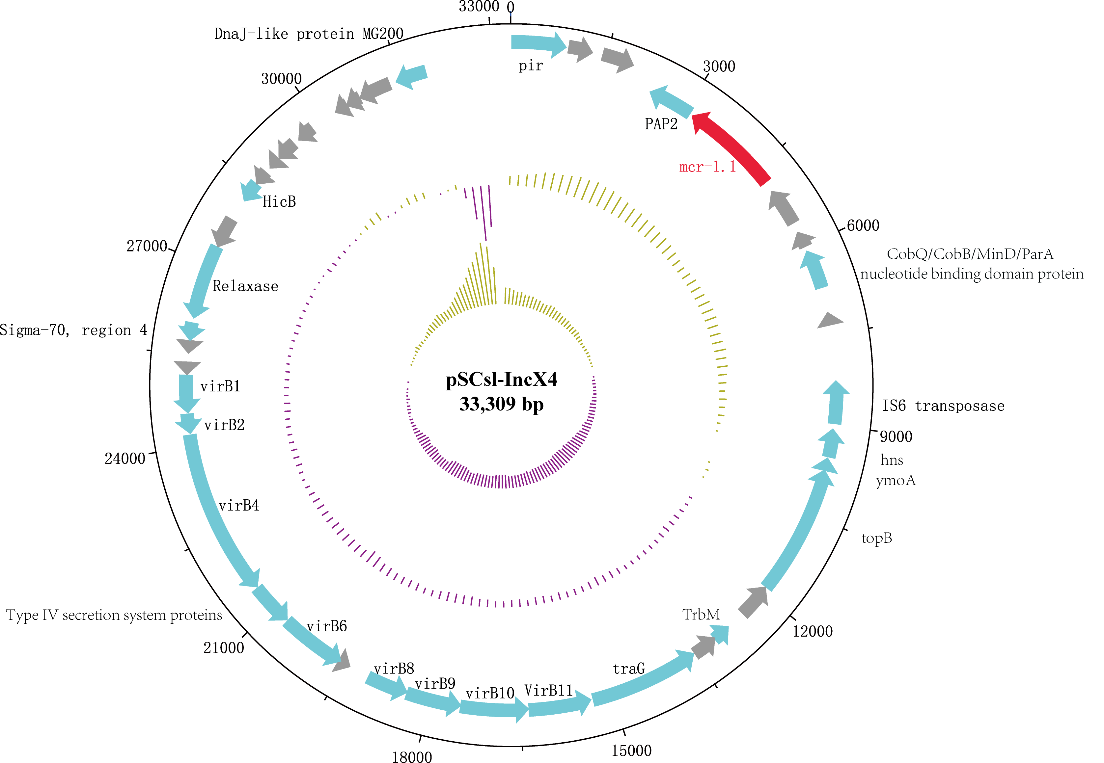


**Figure S1.** Genome map of the plasmid pSCsl19-IncX4. Arrows in the genome map indicate ORFs, which are colored as follows: antibiotic resistance gene (red), other genes, including virulence genes, metabolic genes and so on (blue), and hypothetical protein (gray).
